# Comparing primary caregivers’ reported injury data with routinely recorded injury data to assess predictors of childhood injury

**DOI:** 10.1186/s12874-023-01900-0

**Published:** 2023-04-11

**Authors:** Luam Ghebreab, Bridget Kool, Arier Lee, Susan Morton

**Affiliations:** 1 Section of Epidemiology and Biostatistics, School of Population Health, University of Auckland, 507-1001, 22-30 Park Ave, Auckland, New Zealand; 2 Department of Social and Community Health, School of Population Health, University of Auckland, Auckland, New Zealand

**Keywords:** Data linking, Longitudinal cohort, Child injury, Maternal recall

## Abstract

**Background:**

Linking self-reported data collected from longitudinal studies with administrative health records is timely and cost-effective, provides the opportunity to augment information contained in each and can offset some of the limitations of both data sources. The aim of this study was to compare maternal-reported child injury data with administrative injury records and assess the level of agreement.

**Methods:**

A deterministic linkage was undertaken to link injury-related data from the Growing up in New Zealand (GUiNZ) study to routinely collected injury records from New Zealand’s Accident Compensation Corporation (ACC) for preschool children. The analyses compared: (i) the characteristics of mothers with linked data vs. those without, (ii) injury incidences from maternal recall with those recorded in ACC injury claims, and (iii) the demographic characteristics of concordant and discordant injury reports, including the validity and reliability of injury records from both data sources.

**Results:**

Of all mothers who responded to the injury questions in the GUiNZ study (n = 5836), more than 95% (n = 5637) agreed to have their child’s record linked to routine administrative health records. The overall discordance in injury reports showed an increasing trend as children grew older (9% at 9 M to 29% at 54 M). The mothers of children with discordance between maternal injury reports and ACC records were more likely to be younger, of Pacific ethnicity, with lower educational attainment, and live in areas of high deprivation (p < 0.001). The level of agreement between maternal injury recall and ACC injury record decreased (κ = 0.83 to κ = 0.42) as the cohort moved through their preschool years.

**Conclusions:**

In general, the findings of this study identified that there was underreporting and discordance of the maternal injury recall, which varied by the demographic characteristics of mothers and their child’s age. Therefore, linking the routinely gathered injury data with maternal self-report child injury data has the potential to augment longitudinal birth cohort study data to investigate risk or protective factors associated with childhood injury.

## Introduction

Data linkage is the process of combining two or more records and attempting to match the pair or group of data that fit the same individual [1]. Cohort studies capture information from individuals over an extended period of time. The detailed primary data collected from these participants may not be available in routine health records [2]. However, they have some limitations, including the temporal and financial demands, attrition due to loss-of follow-up [3], the complexity of datasets over a prolonged period of time and the issue of the observational nature of most longitudinal studies [4, 5].

In comparison, large-scale health-related data routinely collected by governments, healthcare providers or insurance companies can be made available in electronic forms where they can be linked over time and across data sources to generate longitudinal records for individuals [4]. In longitudinal research, the opportunity exists to merge the rich primary information acquired from the array of cohort data collection methods with national health care data. These routinely gathered secondary data sources have various administrative purposes and are commonly used in health surveillance to monitor multiple health outcomes to inform efforts to control diseases, actuarial purposes to estimate costs, and burden of disease to improve population health outcomes [5]. Despite the high data coherence and cost-effectiveness, the potential limitation of routine administrative data are completeness due to linkage errors, and the quality of the data [6, 7]. Moreover, routine administrative data frequently has a parsimonious set of variables related to the organisational purpose for which the data was collected and might contain limited exposure variables and key cofounders relevant to a specific area of research [8].

Combining the self-reported longitudinal data from cohort studies with routine healthcare data could assist in counterbalancing the limitations of each data type. In addition to its ability to address the issue of missing data, linking cohort study data with administrative data sources can reduce the respondent burden on cohort study participants to report health outcomes, minimise recall and information biases, and provide an opportunity to validate both data sources [9]. Administrative data linkages in longitudinal studies have been shown to contribute to increasing their data completeness effectively [10]. Given this context, linking routinely gathered health data with longitudinal cohort data has gained popularity over the past few decades with both national and international longitudinal studies [11, 12].

Growing up in New Zealand (GUiNZ) is a contemporary longitudinal birth cohort which aims to map the evidence about the multi-disciplinary determinants of pathways in relation to children’s health and development to inform a prioritised range of evidence-based policy initiatives to improve life-course wellbeing [13] The GUiNZ cohort has been linking longitudinal information gathered directly from families with routinely gathered perinatal, immunisation and other health-related administrative data since its inception in 2009 [14].

There are a number of methodological challenges, ethical issues, and time and resource concerns that need to be considered when undertaking the linkage of cohort data with routinely collected data [15]. The requirement to provide some participant identifiable data to a third party may risk the anonymity and confidentiality that has been given to participants [16]. For this reason, prior to any linkage, a process to match records needs to be negotiated that complies with ethical requirements for the cohort and also for the routine data holder, but nevertheless maximises the likelihood of accurate linkage. Yet, the use of alternative identifiers to link data may produce higher linkage error rates. Safeguarding the data during linkage respects the confidentiality of participants and complies with ethical standards for cohort-specific data sharing, particularly on how the data is linked, and who undertakes the linkage is key to protect anonymity. To mitigate the risk of violating the privacy of participants in linked datasets, pseudo-identifiers are created by data guardians within the GUiNZ research team. Parental consent on behalf of their children for data linkage was sought several times during data collection waves (DCWs) [14].

Childhood injury is a major cause of hospitalisations and deaths in New Zealand[17] and globally and is therefore, a significant public health issue. Ensuring high-quality data provides an essential platform for identifying and prioritising high injury risk groups and planning comprehensive prevention efforts to reduce the burden of injuries [18]. Most research on the incidence and causes of childhood injuries draws on either parental recall or routinely collected health service data. We were interested in utilising the “ecological model of injury across the life course” developed by Hosking et al.[19] to explore multiple factors that surround preschool child injury in New Zealand. We analysed the trajectory of maternal self-reported developmental information across multiple inter-connected disciplinary domains from the GUiNZ study and linked this to ACC (the national no-fault system injury insurance scheme [20]) injury claim data for the cohort. Bringing these two datasets provides a novel way to explore risk factors for child injury in New Zealand; therefore, the aim of this study was to compare maternal-reported child injury data with administrative injury records and assess the level of agreement.

## Methods

A deterministic linkage was undertaken to link injury-related data from the Growing up in New Zealand (GUiNZ) study to routinely collected injury records from New Zealand’s Accident Compensation Corporation (ACC) for preschool children.

### Study participants

The linkage involved children from the GUiNZ longitudinal birth cohort, whose mothers or primary caregivers responded to the injury questions over the preschool period (at nine months [9 M], 24 months [24 M], and 54 months [54 M]) and who had consented for their child’s data to be linked to routine health information. Note, for the purposes of these analyses, the term ‘mother’ has been used to describe the primary caregiver as 99.6% of the mothers at the 9 M DCW and 98.9% at the 54 M DCW were the primary caregivers/ responding during DCWs.

### Data sources

#### Growing up in New Zealand data

GUiNZ is a contemporary and population-relevant longitudinal study of children recruited in pregnancy with expected dates of delivery between April 2009 and March 2010. The cohort was recruited through their pregnant mothers residing within three District Health Board regions (Auckland, Counties-Manukau and Waikato) in New Zealand [21]. The GUiNZ cohort longitudinal information was collected using face-to-face, telephone, and computer-assisted interviews and consent was sought for linkage to routine datasets at different DCWs, including linkage to routine perinatal data from the study’s outset. A total of 6822 pregnant mothers agreed to their children’s participation and completed their antenatal interview. The pregnancies resulted in 6846 children who had survived the first six weeks postnatally (note: 184 were twins or triplets) [22]. Separate interviews have resulted in datasets related to the child and their family and wider environment gathered from interviewing the biological mother and then the mother postnatally (not always the same person) and her social partner at the time. Information has also been accrued via direct observation by trained interviewers during each data DCW. Data on injury were collected at the three time points as previously described.

#### Accident Compensation Corporation data

When medical attention is sought for an injury, an ACC claim form is completed by the patient (or caregiver) and the attending clinician [23]. The information captured by ACC includes injury date, year, context, intention, and provisional diagnosis [23]. ACC data is a valuable source of population-level injury data covering the continuum of injury severity from minor to major injury (including fatal injury).

### Linkage process

This study used a “Master” linkage structure for the purpose of defining the outcome of “unintentional child injury”, where the linkage of GUiNZ cohort participants with an ACC claim is meaningfully interpreted as a cohort participant having an injury [24]. Subsequently, the participant’s injury data is merged into all cohort participants (with exposure data, with and without injury) to analyse the predictors of injury. Commonly, there are two approaches to linking data: probabilistic and deterministic (exact methods) [25]. Probabilistic linkage is a flexible technique used when there is no linkage variable that is precisely the same in both data sets and when additional variables are required to ensure all matches are made but has increased matching errors [26]. To minimise the false-matching and missed-matching rates [27] the deterministic approach was employed using the study participant’s unique National Health identifier (NHI). Individuals’ NHI within the GUiNZ study came from linkage in the perinatal period and with explicit consent. Additional identifiers such as gender and month of birth of the cohort were provided as accessory identifying variables for linkage to check for correct matches in case the NHI was corrupted, for example, in either dataset.

The extracted data from ACC included any injury episodes associated with the GUiNZ cohort of interest from birth to 54 months. A manual review of records was used to determine whether the linked ACC injury data matched with an individual record in the GUiNZ data, by ensuring the date of birth and gender matched. Injury claims that occurred outside the period of interest were excluded.

### Outcome of interest

**Fig. 1:**
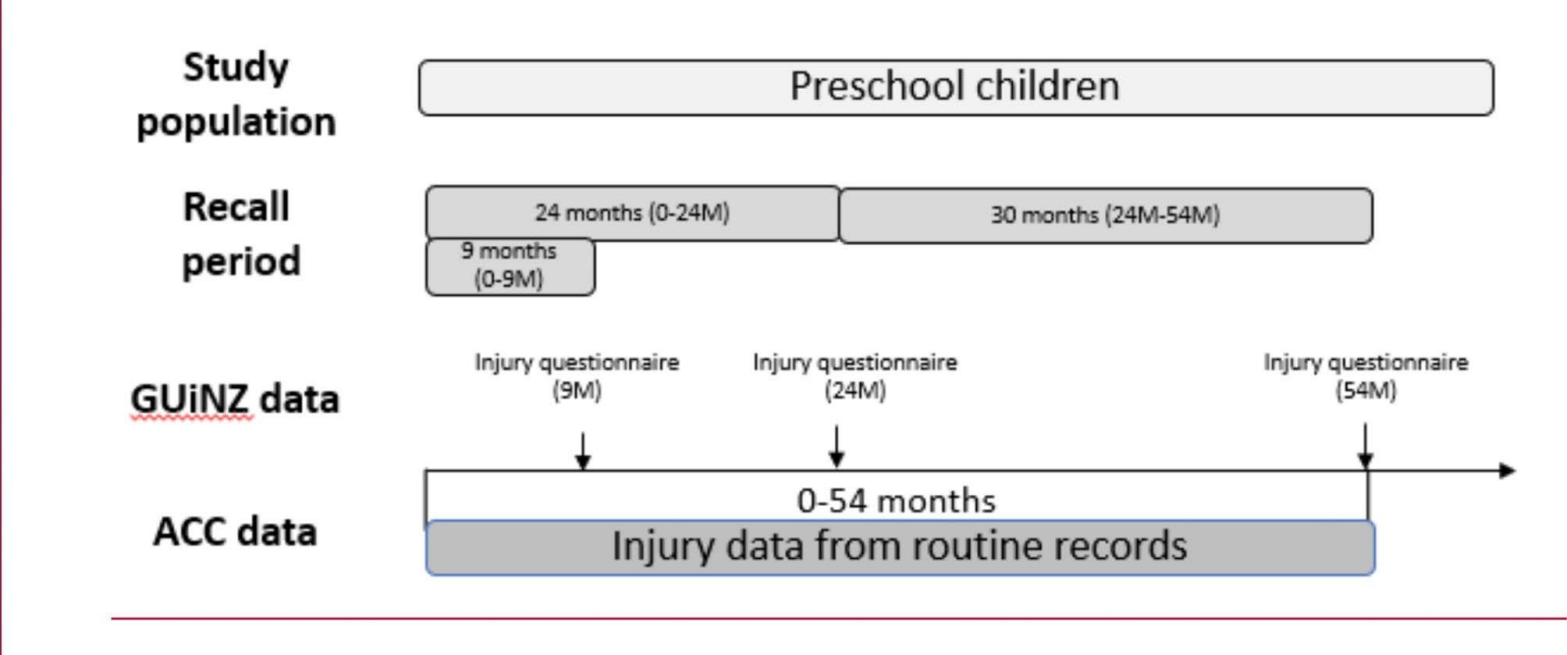
Study population and source of data

The GUiNZ dataset captures a vast array of age- and time-specific wellbeing and developmental information. During the face-to-face interviews at 9 M and 24 M, mothers were asked, “Has your child ever had an accident or injury for which [he/she] was taken to the doctor, health centre, or hospital since birth?“ with “yes” or “no” response options provided (Fig. 1). However, at the 54 M face-to-face interview, mothers were asked the same injury question but only if the child ‘had ever had any injury since [he/she] was two years old’. For the purposes of this study, children whose mothers had responded “yes” or “no” to this question for the time period of interest (i.e. < 5 years of age) were included. Responses such as “Don’t know” and “Refused to answer” from primary caregivers were very infrequent (< 0.01%) and considered missing. In addition to identifying children who sustained an injury, further analysis was conducted on the maternal-reported count of injuries obtained from responses to the question “How many accidents or injuries?“. Note that these questions were asked amongst a set of questions relating to health and wellbeing, which is only one of the six domains that GUiNZ collects information on at each DCW.

Further information was also gathered about a child’s injury but only on a selected injury in any time period that the mother deemed ‘most significant’. For example, at the 24 M DCW, they were asked, “Thinking about the most severe (or only) accident or injury since birth, what sort of accident or injury was it?“, and similarly at the 54 M DCW “Thinking about the most severe (or only) accident or injury since {NAME} was two, what sort of accident or injury was this?“. As a result, each injury sustained by the children in the cohort was unable to be compared. Therefore, only two outcomes were analysed in this study: sustained an injury vs. no injury and the injury frequency.

### Statistical analysis

Descriptive statistical analysis was undertaken to characterise and compare the maternal-reported injury events from GUiNZ data with the injury events for the cohort captured in the ACC claims data. We were interested if the maternal reported injury data compared with ACC claims differed for the three DCWs (0 to 9 months, 0 to 24 months and 24 months to 54 months); therefore, the tables and figures are presented in this way. The level of missingness is reported, including where there was maternal self-reported injury, where there was no corresponding ACC claim or vice-versa, and where variables of interest were not captured.

Pearson’s Chi-square test of significance analysis was undertaken to identify any existing direction of misclassification between the GUiNZ study maternal reports of injury, and the ACC injury records, by reviewing the symmetry of the discrepant dichotomous injury classification. A range of maternal demographic characteristics was explored using cross-tabulation analysis to determine the distribution of mothers’ discordant (underreporting or overreporting ACC injury reports) and concordant results. For the purpose of comparison, the ACC record was used as the gold standard because it is explicitly designed to capture all significant injuries (requiring medical intervention), whereas the GUiNZ questions were not designed to elicit all injuries. Thus, maternally reported child injury validity was evaluated using sensitivity, specificity, and positive and negative predictive values [28]. The degree of reliability was assessed using the prevalence, and bias-adjusted kappa statistic (as kappa is commonly affected by the prevalence of an indicator and level of disagreement) [29] which revealed the percentage of agreement beyond chance between GUiNZ study cohort reporting of childhood injuries by mothers and the ACC captured claims. A non-parametric paired t-test (Wilcoxon signed-rank test) was used to measure the count difference for all matched pairs. Finally, the results were ordered separately to give positive and negative differences [30].

## Results

### GUiNZ-ACC analysis cohort

Completion of the injury-related questions at each of the three DCWs of interest was as follows: 6474 mothers completed the questions at the 9 M DCW, 6321 at the 24 M DCW, and 6141 at the 54 M DCW. Consent to link the routinely collected health records was obtained during child observation of 54 M DCW (N = 5836), out of which 96.6% (n = 5637/5836) agreed to link their data, and 3.4% (n = 199/5836) did not agree. The number of responses to injury questions by mothers and ACC injury reports for those who consented at each DCW is displayed in Table 1. Around 85%, 87% and 92% of those who responded to GUiNZ injury questions agreed to link their data with routinely collected health data at 9 M, 24 M and 54 M DCWs, respectively.

**Table 1:** Consent to linkage rates for mothers in GUiNZ who responded to the injury-related question at each of the three data collection data of interest

Age (DCW)	Mothers responding to the GUiNZ injury questions	Mothers who agreed to data linkage n(%)*	Mothers who did not agree to data linkage n(%)	No response to consent (or missing) to data linkage n(%)
0–9 months	6474	5499(85.0)	192(3.0)	783(12.0)
0–24 months	6321	5511(87.2)	190(3.0)	620(9.2)
24–54 months	6141	5624(91.6)	199(3.2)	318(5.2)
0–54 months	5961	5500(92.3)	190(3.2)	271(4.5)

*Some of the 5637 participants who agreed to link their data during child observation of 54 M DCW did not respond to the injury questions

### Characteristics of mothers in the GUiNZ cohort who answered the injury questions and agreed to data linkage compared with those who did not

Of the 5961 mothers who answered injury questions at both 24 M and 54 M, 92% (n = 5500/5961) agreed to have their data linked, and 8% (n = 461/5961) either did not agree or there was no response recorded to the consent provided (due to attrition or loss to follow-up during the DCW) to link their data (Table 2). Among the 5500 mothers who agreed to have data linked, the majority (56%; n = 3088/5449) were aged between 25 and 34 years, identified as European (62.9%; n = 3448/5483), and reported medium or high levels of socioeconomic deprivation 38% and 36%, respectively. There were significant differences (p < 0.001) in maternal ethnicity between those who agreed to have their data linked and those who did not. Of note, a greater proportion of European mothers answered the injury questions and agreed to data linkage (93.5%; n = 3223/3448) compared with Asian (89.7%; n = 711/793) or Other ethnicities mothers (85.4%; n = 169/198). There was no significant difference between the two groups by maternal age, NZDep, child gender or injury.

**Table 2:** Characteristics of mothers in the GUiNZ cohort who answered the injury questions and agreed to data linkage, compared with those who did not (row %), 0–54 months combined data collection period (N = 5961)

Variables	Total N = 5961	Mothers who answered the injury questions and who agreed to data linkage n = 5500 (92%) n (%)	Mothers who answered the injury questions and who did not agree to or did not respond to data linkage n = 461(8%) n (%)	P-value *
Maternal age				0.818
< 25 years	1034	953 (92.2)	81 (7.8)	
25 to 34	3352	3088 (92.1)	264 (7.9)	
≥ 35	1474	1458 (92.6)	116 (7.4)	
Missing	1	1	0	
Maternal Ethnicity	5943			< 0.001
Māori	773	715 (92.5)	58 (7.5)	
Pacific	731	665 (91.0)	66 (9.0)	
European	3448	3223 (93.5)	225 (6.5)	
Asian	793	711 (89.7)	82 (10.3)	
Others	198	169 (85.4)	29 (14.6)	
Missing	18	17	1	
NZDep**				0.209
Low (1–3)	1586	1474 (92.9)	112 (7.1)	
Medium (4–7)	2263	2071 (91.5)	192 (8.5)	
High (8–10)	2109	1953 (92.6)	156 (7.4)	
Missing	3	2	1	
Child gender				
Male	3067	2834 (92.4)	233 (7.6)	0.684
Female	2894	2666 (92.1)	228 (7.9)	
Injury				
Yes	3026	2784 (92.0)	242 (8.0)	0.439
No	2935	2716 (92.5)	219 (7.5)	

*P-value for Pearson’s Chi-Square

** NZDep: New Zealand Deprivation Index 2006

### Distribution of concordant reports of injury comparing maternal reports with ACC data

**Fig. 2:**
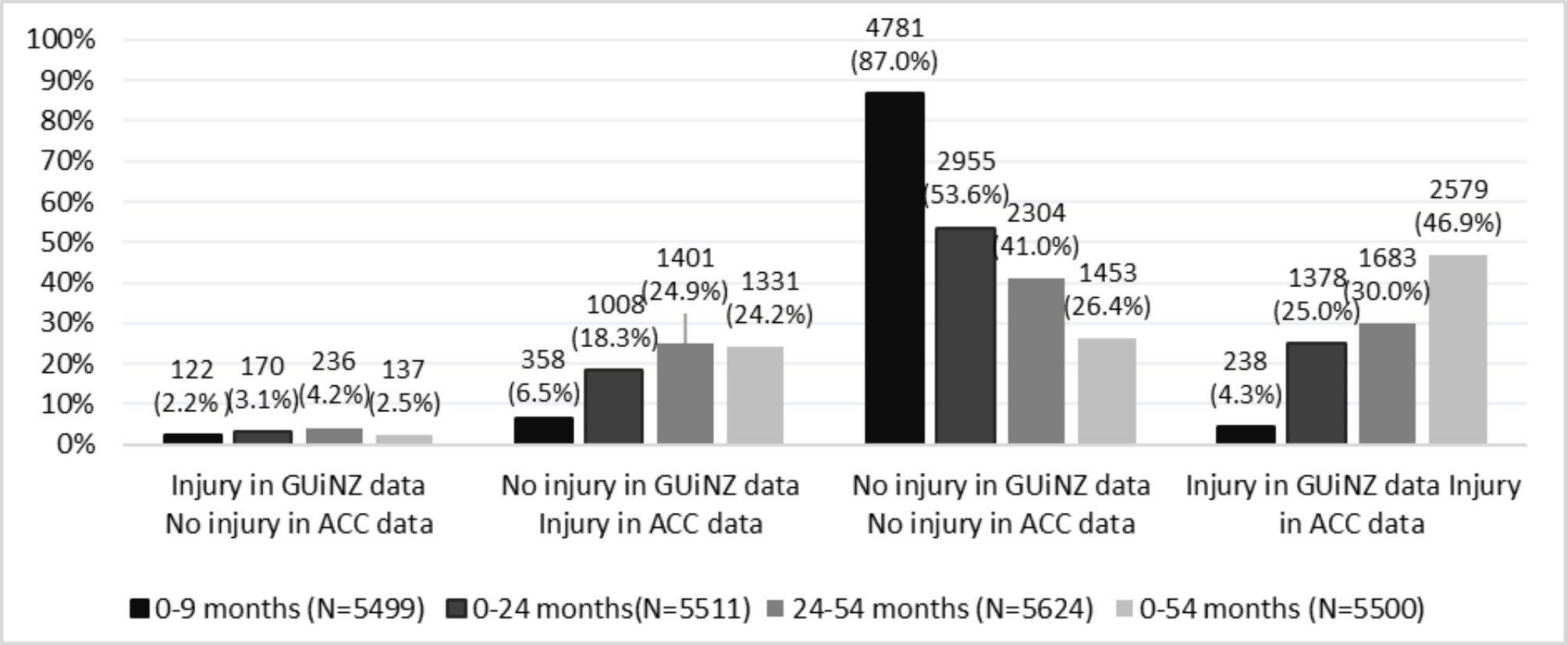
Frequency of injury reports from ACC and maternal reports from GUiNZ

The frequency of instances where mothers reported injuries in the GUiNZ data, but the injury did not appear in ACC data during the same period of time (not necessarily the same injury event) increased from 122 at the 9 M DCW, 170 at 24 M, to 236 at 54 M of age (Fig. 2). The number of injuries reported to ACC but where mothers had not reported the injury in GUiNZ DCWs increased as children grew older, from 358 to 9 M to 1401 at 54 M of age.

Similarly, the frequency of the overall discordant injury reports (mothers who reported injuries, but no injury reported in ACC data or mothers who did not report injury, but injury/injuries reported in ACC data) generally showed an upward trend as children’s age increased, varying from 9% (n = 495/5499) to 29% (n = 1637/5624) from 9 M to 54 M, respectively (Table 3). This discordance was more apparent among mothers who were younger at the time their child was born (< 25 years old), of Māori and Pacific ethnicity (p < 0.001), with lower educational attainment (p < 0.001), and with consistently high deprivation levels (p < 0.001) throughout the DCWs. For pregnancy-related characteristics, mothers with unplanned pregnancies were more likely to report discordance at all time points (p < 0.001). Mothers of children with more than three siblings were significantly more likely to report discordant injury reports at 24 M and 54 M (p = 0.001). However, parity was not associated with injury reports’ concordance at 9 M (p = 0.366).

**Table 3:** Comparison of concordant vs. discordant reports by sociodemographic characteristics at 9-months, 24 months and 54 months (row percentage%)

	0-9 m			0-24-months			24-54-months data collection wave		
Variables	Concordant * **5142 (91%)**	**Discordant** ^**#**^ **495(9%)**	**P-value**	**Concordant** **4333(79%)**	**Discordant** **1178(21%)**	**P-value**	**Concordant** **3987(71%)**	**Discordant** **1637(29%)**	**P-value**
Maternal age			< 0.001			< 0.001			0.003
< 25 years	830 (87.7)	116(12.3)		674(70.6)	280(29.4)		659(66.9)	326(33.1)	
25 to 34	2828 (91.4)	265(8.6)		2453(79.3)	639(20.7)		2245(71.0)	915(29.0)	
>=35	1358 (93.2)	99(6.8)		1204(82.3)	258(17.7)		1080(73.2)	396(26.8)	
Maternal ethnicity			< 0.001			< 0.001			< 0.001
Māori	627 (87.9)	86(12.1)		522(73.0)	193(27.0)		503(67.8)	239(32.2)	
Pacific	567 (85.0)	100(15.0)		442(66.4)	224(33.6)		418(59.1)	289(40.9)	
European	2988(92.9)	227(7.1)		2660(82.4)	570(17.6)		2441(75.3)	801(24.7)	
Asian	668(93.1)	50(6.9)		554(77.9)	157(22.1)		492(66.1)	252(33.9)	
Others	155(91.7)	14(8.3)		140(82.3)	30(17.7)		117(68.8)	53(31.2)	
Maternal Education			< 0.001			< 0.001			< 0.001
No secondary school	288(84.5)	53(15.5)		239(69.9)	103(30.1)		226(62.6)	135(37.4)	
Secondary/NCEA1-4	1103(90.4)	117(9.6)		924(75.8)	295(24.2)		846(67.1)	414(32.9)	
Diploma/Trade cert./NCEA 5–6	1530(91.5)	142(8.5)		1306(77.9)	370(22.1)		1217(71.3)	489(28.7)	
Bachelor’s deg.	1240(92.3)	103(7.7)		1096(81.5)	249(18.5)		1003(73.6)	359(26.4)	
Higher degree	845(93.2)	62(6.8)		758(83.0)	155(17.0)		683(74.2)	237(25.8)	
NZDep**			< 0.001			< 0.001			< 0.001
Low (1–3)	1376(93.5)	95(6.5)		1208(81.7)	270(18.3)		1091(73.4)	396(26.6)	
Medium (4–7)	1918(92.8)	149(7.2)		1695(81.8)	378(18.2)		1550(73.8)	550(26.2)	
High (8–10)	1721(87.9)	236(12.1)		1427(73.0)	529(27.0)		1343(66.1)	690(33.9)	
Parity			0.366						-
First	2084(90.9)	209(9.1)		-	-		-	-	
Subsequent	2927(91.6)	269(8.4)		-	-		-	-	
Sibling 16 M/54 M						0.001			0.001
0	-	-	-	1709(80.7)	409(19.3)		459(68.8)	208(31.2)	
1	-	-	-	1495(78.6)	407(21.4)		1737(73.0)	642(27.0)	
2	-	-	-	676(77.7)	194(22.3)		1037(71.7)	410(28.3)	
3+	-	-	-	453(72.9)	168(27.1)		754(66.7)	377(33.3)	
Planned pregnancy			< 0.001			< 0.001			< 0.001
Yes	3163(92.4)	259(7.6)		2781(81.2)	642(18.8)		2526(72.7)	947(27.3)	
No	1832(89.5)	215(10.5)		1528(74.2)	531(25.8)		1439(67.8)	682(32.2)	
Maternal Birthplace			0.345			0.023			< 0.001
New Zealand	3340(91.5)	309(8.5)		2905(79.4)	754(20.6)		2712(73.1)	998(26.9)	
other	1611(90.8)	164(9.2)		1363(76.7)	414(23.3)		1219(66.4)	616(33.6)	

### Comparing the count of maternally reported injury data with ACC-reported injury data

**Fig. 3:**
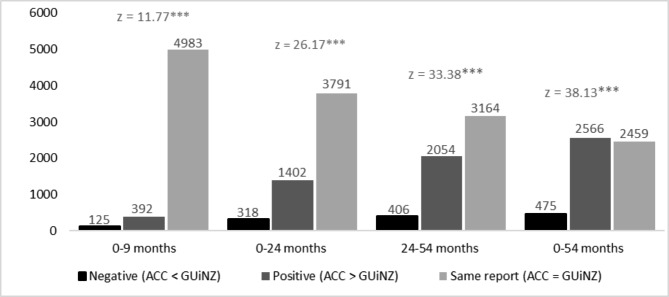
Wilcoxon signed-rank paired test between ACC and GUiNZ count injury data

Across all age groups, in both the GUiNZ captured injury data and the ACC injury data, more than 80% of children sustained one to three injuries from birth to 54 M (Table 4). Overall, there were more positive differences among the matching injury count pairs. The positive differences increase as the age of children increases, indicating that the maternal underreporting of child injuries during GUiNZ DCWs increases as the child gets older. At 0-9 M, there were 392 positive differences and 125 negative differences between the matched injury count pairs (z = 11.766, p = < 0.001), this increased to 2054 positive and 406 negative count differences at 24 M-54 M (z = 33.38, p < 0.001) (Fig. 3).

**Table 4:** Frequency of child injury from the maternal report in GUiNZ compared with ACC data reports (Column %)

Count of injury	0–9 months		0–24 months		24–54 months		0–54 months	
	GUiNZ N = 360 n(%)*	ACC N = 596 n(%)	GUiNZ N = 1548 n(%)	ACC N = 2386 n(%)	GUiNZ N = 1919 n(%)	ACC N = 3084 n(%)	GUiNZ N = 2716 n(%)	ACC N = 3910 n(%)
1–3	353 (98.0)	592 (99.0)	1498 (96.8)	2266 (95.0)	1842 (96.0)	2815 (91.3)	2484 (91.5)	3205 (82.0)
4–6	5 (1.4)	4 (1.0)	44 (2.8)	102 (4.3)	57 (3.0)	229 (7.4)	189 (7.0)	556 (14.2)
7–9	-	-	2 (0.1)	15 (0.6)	7 (0.4)	30 (1.0)	21 (0.8)	105 (2.7)
10+	2 (0.6)	-	4 (0.3)	3 (0.1)	13 (0.7)	10 (0.3)	22 (0.8)	44 (1.1)

*Percentage is calculated from the total number of sustained injuries only

*** P < 0.001; *percentage is calculated from the total number of sustained injuries only; Negative (ACC- GUiNZ injury count less than zero); Positive (ACC - GUiNZ = injury count greater than zero); Same (GUiNZ -ACC injury count is zero)

*PABAK (κ): Prevalence adjusted bias-adjusted kappa; all κ = P < 0.001*

### Validity and reliability of child injury reports in the GUiNZ and ACC records

**Fig. 4:**
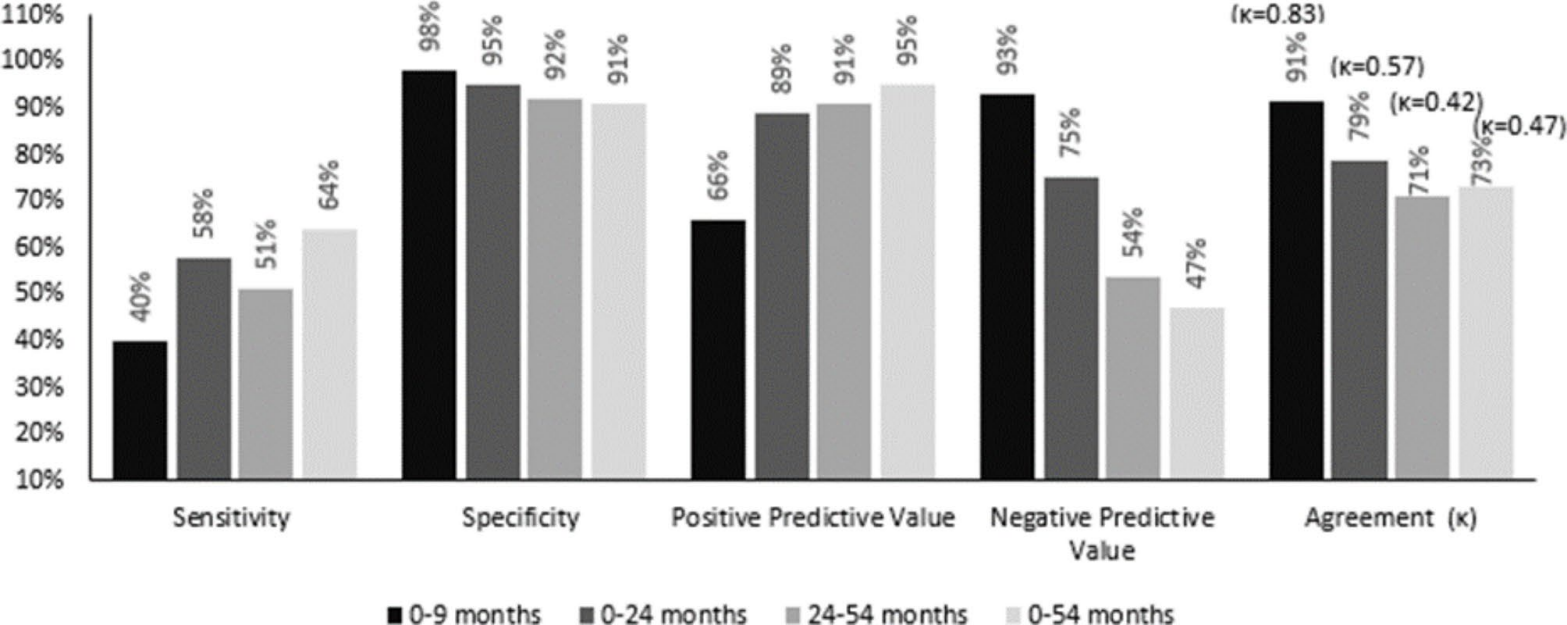
Sensitivity, Specificity, PPV, NPV, agreement comparison of GUiNZ and ACC injury frequency at different data collection waves

In general, mothers tended to underreport injury events, resulting in lowered sensitivity, high specificity, and generally lower kappa agreement (Fig. 4). At 9 M, the percentage of children whose mothers recalled they were injured, and where there was a corresponding ACC record (sensitivity = 40%) was lower than the percentage of children whose mothers reported they did not sustain injury and there was no corresponding ACC record (specificity = 98%). The level of agreement between maternal injury recall and ACC injury record was above 90% at 9 M with high-adjusted Kappa agreement (κ = 0.83) between the two data resources, but despite relatively high levels of agreement (79% and 71% at 24 M and 54 M respectively), the adjusted Kappa level remained moderate (κ = 0.42–0.57).

## Discussion

The aim of this paper was to compare the childhood injury data from maternal recall captured in a longitudinal birth cohort with routinely gathered injury data and assess the level of agreement between these data sources. The comparative injury analysis was undertaken among children whose mothers agreed to link their data. Mothers reported fewer injury incidences overall, and as the children grew older, more injuries were captured in ACC records, and the injury frequency gap between maternal recall and ACC injury claims widened. The discordance between injury reports was more common among younger mothers, those who identified themselves as of Pacific ethnicity, those with less completed formal education, and those living in higher deprivation areas.

The vast majority of mothers consented to the linkage of GUiNZ data to routinely collected health records (including ACC records). MMaternal agreement to data linkage varied considerably by ethnicity of the primary caregivers. Consistent with findings from a UK cohort study [31–33], higher proportions of Asian mothers and those from other minor ethnicities did not consent to data linkage in the present study, and therefore were excluded from the analysis. Some researchers note that the under-representation of minority groups in all health-related research is at the initial recruitment stage due to language and culture barriers, concerns of adverse consequences, and mistrust of the study [34].

In the GUiNZ study, the initial demographic characteristics of recruited participants were comparable with the New Zealand population; sample size calculations were also designed to accommodate the anticipated attrition rate [13]. It is crucial in countries with a multi-ethnic population like New Zealand that longitudinal studies adopt approaches that utilise culturally appropriate frameworks to minimise participation differences [35]. However, the most plausible explanation for the variability by ethnicity between those who consented to link their data and those who did not is likely due to the timing of the consent process. The consent was obtained when the cohort were 54 M old. The number of GUiNZ participants included in this comparative analysis is reduced compared to the initial number of included cohorts due to attrition between 9 M and 54 M, which is common in longitudinal studies [36]. Among the GUiNZ cohort, attrition tends to be biased because the highest loss of participants has occurred in groups who reside in highly disadvantaged areas and among those who identify with an ethnicity other than New Zealand European [37].The time interval since exposure and the degree of detail required, the significance of the events, the interviewing methods used, the influence of social desirability and the individual’s characteristics (such as age, socioeconomic status, and academic and health literacy level) have been identified as leading factors that contribute to recall bias in epidemiological research [38]. This study found that the proportion of discordant injury reports was significantly higher among young mothers (< 25 years of age), those identifying as Pacific or Māori, mothers with lower educational attainment, and those living in high deprivation levels. These findings align with those from other international studies [39–41]. Similar to the findings of this study, a study of validation on maternal recall on breastfeeding duration in the United States found mothers with higher education (college and beyond) had the highest level of agreement between recorded and recall-related to breastfeeding history [42].

In the present study, mothers identified as Pacific had the highest discordance rate at 24 M (33%). Language barriers or misinterpretation due to cross-cultural communication inherently influence the data quality and the analytic approach in terms of reliability and validity in observational studies. It is essential to ensure rigorous survey instrument translation methods to minimise biases of participants’ responses [43]. However, a New Zealand study comparing the injury reports from the Pacific Islands Families (PIF) cohort study and National Minimum Dataset reported no systemic under-reporting of injury events by the PIFs, suggesting low recall bias [12]. Only 62% of the mothers reported they spoke English fluently within the PIF study. In contrast, in the GUiNZ study, one-third of parents of children are born outside of New Zealand, and English is the primary language for 80% of the households, while the remainder communicate in a range of other languages. Thus, the potential explanation for the discordance of injury reports could possibly be due to the nature of the questions asked and the comparability with linked data.

This study found that mothers reported significantly lower injury events during the GUiNZ DCWs compared to the ACC reports, despite the modest to high level of agreeability between both data sources ranging from 71 to 91% between different DCWS. To accurately assess any bias, the broader context of data quality issues, such as a lack of standardised data definitions and inconsistent questionnaires or forms that may lead to errors between the source data, should be discussed and addressed accordingly [15]. In this study, discrepancies in injury reports between these two data sources could also be due to the nature of the injury questions asked. Despite trained interviewers who sought to facilitate the appropriateness and comprehensiveness of the questions during DCWs, the mother’s perception of an injury might vary.

Some injuries reported by mothers were not captured in the ACC data, which highlights the limitation of the “Master” linkage structure using routinely collected disease registry data event-based records [4, 24]. As such, there is the implication of two types of injury status misclassification: a missed match where a cohort participant is inaccurately classified as being injury-free and/or a false match where a participant from the cohort without injury is linked with the ACC registry [44]. ACC contains only records generated as a result of an event and does not provide information on children who have not experienced injury.

The study needs to be considered in light of several limitations. ACC claims data provides detailed information regarding the nature of injuries sustained, the date of the event, and the scene of injury for any injuries where treatment was provided. However, no information regarding the severity of injury is available. Data linkage was only possible among mothers who consented to link their child’s data to routinely collected data. Lack of access to injury data from non-consenting mothers means the injury difference will remain unmapped; however, given the high agreement rate to consent, this risk remains low.

The GUiNZ data captured mothers’ recall of any injury events where medical treatment was sought and what they recall and perceive as the most severe of these. Note that no information regarding the dates of injury events was captured in the GUiNZ data. For these reasons, the individual injury events between both data sources were not matched. The present study only dichotomised the primary outcomes of interest as injury versus no injury and the frequency of injuries. Comparing the types and hospitalisations of injuries left unreported or not received from the ACC claim record was not also achievable.

The maternal reported data was subject to bias due to underreporting by various sociodemographic characteristics. However, despite its limitations, the maternal injury data can provide different perspective on the circumstances of injury. For example, in this study the maternal report had additional information on severity and history of hospitalisation. The findings of this study have demonstrated that linking longitudinal GUiNZ cohort study data– that include information on a range of known and potential risk factors for child injury ranging from their immediate family environments to their wider societal context, over time – with routine injury data from ACC can help provide a more comprehensive picture of injury than from individual sources alone. This augmented information can be used to inform public health approaches to injury prevention. Linkage is deemed a robust and established measurement tool, but researchers should consider the limitations and challenges in the linkage process and the quality of both data sources [45].

The ability to create a comprehensive linked injury dataset with minimum bias has broad implications for public health planning. Linking survey data to routinely collected data can access a variety of additional injury information to support injury prevention efforts and enhance existing evidence to inform policy initiatives. Data linkage also provides an opportunity to minimise participant respondent burden, and the associated cost of recruiting participants for research [46]. Linkage of cohort study data to routinely collected data may also provide the opportunity to follow patients lost to follow-up consent has been obtained to do so.

## Conclusion

This study has demonstrated that the reliability of maternal injury recall differed by demographic characteristics, and the accuracy reduced as the cohort moved through their preschool years. Therefore, augmenting longitudinal birth cohort study data, by linking with routinely collected injury data has a potential to, allow a broader understanding of the factors contributing to injury risk among children. Conducting studies that compare injury data sources can also inform the adoption of appropriate measuring tools in injury-related observational studies.

## Data Availability

The data that support the findings of this study are available from the Growing Up in New Zealand study [please see https://www.growingup.co.nz/access-growing-data, contact data access co-ordinator at “dataaccess@growingup.co.nz”], but restrictions apply to the availability of these data, which were used under license for the current study, and so are not publicly available. The data are not publicly available due to containing information that could compromise research participant privacy and consent.

## List of Abbreviations

ACC: Accident Compensation Corporation
GUiNZ: Growing up in New Zealand
DCW: Data Collection Wave
NHI: National Health identifier
NZDep: New Zealand Deprivation Index 2006
PIF: Pacific Islands Families

## References

[1] Belin TR, Rubin DB (1995). A method for calibrating false-match rates in record linkage. J Am Stat Assoc.

[2] Peterson L, Harbeck C, Moreno A (1993). Measures of children’s injuries: self-reported Versus maternal-reported events with temporally proximal Versus delayed reporting. J Pediatr Psychol.

[3] Caruana EJ, Roman M, Hernández-Sánchez J, Solli P (2015). Longitudinal studies. J Thorac Dis.

[4] Jorm L (2015). Routinely collected data as a strategic resource for research: priorities for methods and workforce article history. Public Health Res Pract.

[5] Thacker SB, Berkelman RL (1988). Public health surveillance in the United States. Epidemiol Rev.

[6] Harron K, Wade A, Gilbert R, Muller-Pebody B, Goldstein H. Evaluating bias due to data linkage error in electronic healthcare records. BMC Med Res Methodol. 2014;14. 10.1186/1471-2288-14-36 PMC4015706 24597489

[7] Bohensky MA, Jolley D, Sundararajan V, Evans S, Pilcher D, Scott I (2010). Data linkage: a powerful research tool with potential problems. BMC Health Serv Res.

[8] Sinha S, Peach G, Poloniecki JD, Thompson MM, Holt PJ (2013). Studies using English administrative data (Hospital Episode Statistics) to assess health-care outcomes–systematic review and recommendations for reporting. Eur J Public Health.

[9] Mountain JA, Nyaradi A, Oddy WH, Glauert RA, de Klerk NH, Straker LM et al. Data linkage in an established longitudinal cohort: The Western Australian Pregnancy Cohort (Raine) Study. Public Health Res Pract. 2016;26. 10.17061/phrp2631636 27421348

[10] Holman CDA, Bass AJ, Rosman DL, Smith MB, Semmens JB, Glasson EJ (2008). A decade of data linkage in western Australia: strategic design, applications and benefits of the WA data linkage system. Aust Health Rev.

[11] Tingay KS, Bandyopadhyay A, Griffiths L, Akbari A, Brophy S, Bedford H et al. Record linkage to enhance consented cohort and routinely collected health data from a UK birth cohort. Int J Popul Data Sci. 2019;4. 10.23889/ijpds.v4i1.579 PMC8142967 34095526

[12] Robertson H, Schluter PJ, Sundborn G (2011). Reliability and validity of maternal recall of injuries in Pacific children: findings from the Pacific Islands families study. Pac Health Dialog.

[13] Morton SMB, Atatoa carr PE, Grant CC, Robinson EM, Bandara DK, Bird A (2013). Cohort profile: growing up in new zealand. Int J Epidemiol.

[14] Growing up in New Zealand. External Data Release 2018 Reference and Process User Guide. 2018.

[15] Harron K, Dibben C, Boyd J, Hjern A, Azimaee M, Barreto ML (2017). Challenges in administrative data linkage for research. Big Data Soc.

[16] Aitken M, de St Jorre J, Pagliari C, Jepson R, Cunningham-Burley S (2016). Public responses to the sharing and linkage of health data for research purposes: a systematic review and thematic synthesis of qualitative studies. BMC Med Ethics.

[17] Duncanson M, Oben G, Adams J, Wicken A, Morris S, Richardson G, et al. Health and wellbeing of under-15 year olds in Aotearoa 2018. Dunedin; 2019.

[18] Mitchell RJ, Cameron CM, Bambach MR (2014). Data linkage for injury surveillance and research in Australia: perils, pitfalls and potential. Aust N Z J Public Health.

[19] Hosking J, Ameratunga S, Morton S, Blank D. A life course approach to injury prevention: A “lens and telescope” conceptual model.BMC Public Health. 2011;11. 10.1186/1471-2458-11-695 PMC3177801 21899775

[20] Accident Compensation Corporation. Statement of Intent 2018–2022 Accident Compensation Corporation Te Kaporeihana Āwhina Hunga Whara. 2018.

[21] Morton SM, Ramke J, Kinloch J, Grant CC, Atatoa Carr P, Leeson H (2015). Growing up in New Zealand cohort alignment with all New Zealand births. J Public Health (Bangkok).

[22] Morton SMB, Atatoa Carr PE, Grant CC, Lee AC, Bandara DK, Mohal J et al. Growing Up in New Zealand: A longitudinal study of New Zealand children and their families. Report 2: Now we are born. 2012.

[23] ACC. IDI data dictionary. ACC injury claims data.New Zealand. Statistics New Zealand Accident Compensation Corporation. 2015.

[24] Doidge JC, Harron KL (2019). Reflections on modern methods: linkage error bias. Int J Epidemiol.

[25] Newcombe HB, Kennedy JM, Axford SJ, James AP. Automatic linkage of vital records. Science (1979). 1959;130:954–9. 10.1126/science.130.3381.954 14426783

[26] Clark DE (2004). Practical introduction to record linkage for injury research. Inj Prev.

[27] Harron K, Gilbert R, Cromwell D, van der Meulen J (2016). Linking data for mothers and babies in De-Identified Electronic Health Data. PLoS ONE.

[28] Krakowiak P, Walker CK, Tancredi DJ, Hertz-Picciotto I (2015). Maternal Recall Versus Medical Records of metabolic conditions from the prenatal period: a validation study. Matern Child Health J.

[29] Byrt T, Bishop J, Carlin JB (1993). Bias, prevalence and kappa. J Clin Epidemiol.

[30] Proudfoot JA, Lin T, Wang B, Tu XM (2018). Tests for paired count outcomes biostatistical methods in psychiatry. Gen Psychiatr.

[31] Knies G, Burton J (2014). Analysis of four studies in a comparative framework reveals: Health linkage consent rates on british cohort studies higher than on UK household panel surveys. BMC Med Res Methodol.

[32] Smart A, Harrison E. The under-representation of minority ethnic groups in UK medical research. http://dx.doi.org/101080/1355785820161182126. 2016;22:65–82. 10.1080/13557858.2016.1182126 27174778

[33] al Baghal T. Obtaining data linkage consent for children: factors influencing outcomes and potential biases. http://dx.doi.org/101080/1364557920151064635. 2015;19:623–43.

[34] Quay TAW, Frimer L, Janssen PA, Lamers Y (2017). Barriers and facilitators to recruitment of South Asians to health research: a scoping review. BMJ Open.

[35] Carter K, Shaw; C, Hayward  M, Blakely  T, Shaw C, Hayward M (2010). Understanding the determinants of consent for linkage of administrative health data with a longitudinal survey. New Z J Social Sci Online.

[36] Young AF, Powers JR, Bell SL (2006). Attrition in longitudinal studies: who do you lose?. Aust N Z J Public Health.

[37] Morton SMB, Marks EJ, Grant CC, Underwood L, Walker CG, Fa’alili-Fidow J, et al. Growing up in New Zealand: a longitudinal study of New Zealand children and their families. Transition to school. Auckland; 2018.

[38] Coughlin SS (1990). Recall bias in epidemiologic studies. J Clin Epidemiol.

[39] Hu Y, Chen Y, Wang Y, Liang H. Validity of Maternal Recall to Assess Vaccination Coverage: Evidence from Six Districts in Zhejiang Province, China.Int J Environ Res Public Health. 2019;16. 10.3390/ijerph16060957 PMC6466224 30889780

[40] D’Souza-Vazirani D, Minkovitz CS, Strobino DM (2005). Validity of maternal report of Acute Health Care Use for Children younger than 3 years. Arch Pediatr Adolesc Med.

[41] Tate AR, Dezateux C, Cole TJ, Davidson L, Hockley C, Calderwood L (2005). Factors affecting a mother’s recall of her baby’s birth weight. Int J Epidemiol.

[42] Amissah EA, Kancherla V, Ko YA, Li R (2017). Validation study of maternal Recall on Breastfeeding Duration 6 years after Childbirth. J Hum Lact.

[43] Squires A, Sadarangani T, Jones S (2020). Strategies for overcoming language barriers in research. J Adv Nurs.

[44] Harron K, Doidge JC, Goldstein H (2020). Assessing data linkage quality in cohort studies. Ann Hum Biol.

[45] Jutte DP, Roos LL, Brownell MD (2011). Administrative record linkage as a Tool for Public Health Research. Annu Rev Public Health.

[46] Morgan K, Page N, Brown R, Long S, Hewitt G, del Pozo-Banos M (2020). Sources of potential bias when combining routine data linkage and a national survey of secondary school-aged children: a record linkage study. BMC Med Res Methodol.

